# Versatility of Transcranial Magnetic Stimulation: A Review of Diagnostic and Therapeutic Applications

**DOI:** 10.3390/brainsci16010101

**Published:** 2026-01-17

**Authors:** Massimo Pascuzzi, Nika Naeini, Adam Dorich, Marco D’Angelo, Jiwon Kim, Jean-Francois Nankoo, Naaz Desai, Robert Chen

**Affiliations:** 1 Krembil Research Institute, Toronto Western Hospital, University Health Network, University of Toronto, Toronto, ON M5T 2S8, Canada; massimo.pascuzzi@uhn.ca (M.P.); nika.sajadinaeini@uhn.ca (N.N.); adam.dorich@uhn.ca (A.D.); jiwon.kim3@uhn.ca (J.K.); jean-francois.nankoo@uhn.ca (J.-F.N.); naaz.desai@uhn.ca (N.D.); 2 Division of Neurology, Department of Medicine, University of Toronto, Toronto, ON M5S 1A8, Canada

**Keywords:** transcranial magnetic stimulation (TMS), repetitive transcranial magnetic stimulation (rTMS), diagnostic, therapeutic, movement disorders, cognitive disorders, review

## Abstract

Transcranial magnetic stimulation (TMS) is a non-invasive neuromodulation technique that utilizes magnetic fields to induce cortical electric currents, enabling both the measurement and modulation of neuronal activity. Initially developed as a diagnostic tool, TMS now serves dual roles in clinical neurology, offering insight into neurophysiological dysfunctions and the therapeutic modulation of abnormal cortical excitability. This review examines key TMS outcome measures, including motor thresholds (MT), input–output (I/O) curves, cortical silent periods (CSP), and paired-pulse paradigms such as short-interval intracortical inhibition (SICI), short-interval intracortical facilitation (SICF), intracortical facilitation (ICF), long interval cortical inhibition (LICI), interhemispheric inhibition (IHI), and short-latency afferent inhibition (SAI). These biomarkers reflect underlying neurotransmitter systems and can aid in differentiating neurological conditions. Diagnostic applications of TMS are explored in Parkinson’s disease (PD), dystonia, essential tremor (ET), Alzheimer’s disease (AD), and mild cognitive impairment (MCI). Each condition displays characteristic neurophysiological profiles, highlighting the potential for TMS-derived biomarkers in early or differential diagnosis. Therapeutically, repetitive TMS (rTMS) has shown promise in modulating cortical circuits and improving motor and cognitive symptoms. High- and low-frequency stimulation protocols have demonstrated efficacy in PD, dystonia, ET, AD, and MCI, targeting the specific cortical regions implicated in each disorder. Moreover, the successful application of TMS in differentiating and treating AD and MCI underscores its clinical utility and translational potential across all neurodegenerative conditions. As research advances, increased attention and investment in TMS could facilitate similar diagnostic and therapeutic breakthroughs for other neurological disorders that currently lack robust tools for early detection and effective intervention. Moreover, this review also aims to underscore the importance of maintaining standardized TMS protocols. By highlighting inconsistencies and variability in outcomes across studies, we emphasize that careful methodological design is critical for ensuring the reproducibility, comparability, and reliable interpretation of TMS findings. In summary, this review emphasizes the value of TMS as a distinctive, non-invasive approach to probing brain function and highlights its considerable promise as both a diagnostic and therapeutic modality in neurology—roles that are often considered separately.

## 1. Introduction

**Figure 1:**
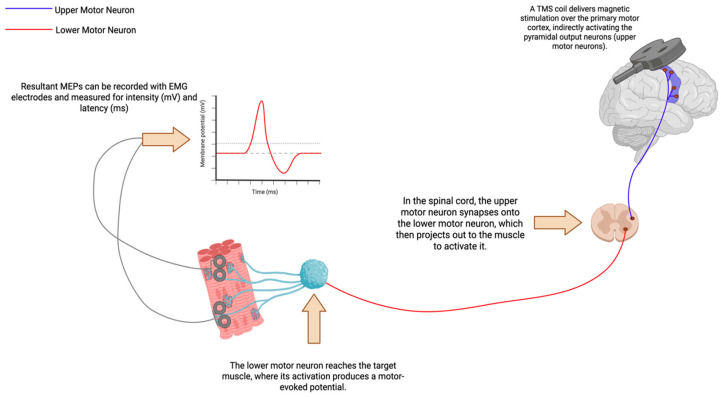
Schematic of TMS: from stimulation to motor effect. This diagram illustrates TMS applied over the M1 using a figure-of-eight coil. The stimulation indirectly activates upper motor neurons in the corticospinal pathway, which in turn activate lower motor neurons in the spinal cord, ultimately causing a contraction in the target muscle. The resulting muscle response is recorded as a motor-evoked potential using EMG electrodes.

TMS is a non-invasive, safe, and painless neurophysiological technique used to stimulate or modulate specific cortical targets in the central nervous system (CNS) [1]. First introduced in the 1980s, TMS is based on Faraday’s law of electromagnetic induction: a brief, high-intensity electrical current is discharged into a TMS coil, generating a strong, time-varying magnetic field perpendicular to the coil [2]. This magnetic field penetrates the scalp and skull without significant attenuation and induces an electric field in the underlying brain tissue [1,3,4]. The induced electric currents depolarize neurons, generating action potentials that can be measured via electroencephalography (EEG) or motor evoked potentials (MEPs), or by indirectly using functional magnetic resonance imaging (fMRI) (Figure 1) [5]. When applied to the primary motor cortex (M1), TMS activates the pyramidal output neurons, producing descending corticospinal volleys, resulting in MEPs [6]. TMS’ effects depend on factors such as the coil type (focal figure-of-eight vs. non-focal circular), pulse waveform (monophasic vs. biphasic), number of pulses (e.g., paired-pulse or triple-pulse protocols), stimulation intensity (subthreshold vs. suprathreshold), and the direction of induced cortical currents, all of which determine the specific neuronal populations activated [7,8,9,10,11,12,13,14]. Nonetheless, a foundational understanding of the core protocols and mechanisms is essential for contextualizing their relevance within this review. Single-pulse, paired-pulse, and the less frequently used triple-pulse TMS protocols elicit immediate sensory or motor responses and are primarily utilized to evaluate the conduction efficiency of neural pathways. In contrast, repetitive TMS (rTMS) induces longer-lasting changes in cortical excitability through synaptic plasticity, presenting potential therapeutic benefits for conditions characterized by disrupted cortical excitability [15]. The relevant outcome measures of TMS, along with its diagnostic utility, will be discussed in more detail below.

Although TMS was initially developed as a diagnostic technique in neurology, it has rapidly gained clinical utility across a wide range of neurological and psychiatric disorders. Diagnostic applications are continuously expanding, with threshold tracking and TMS-EEG emerging as powerful tools for detecting cortical biomarkers and mapping motor cortex function, essential in the preoperative planning of brain tumor surgeries [16,17,18,19,20]. In addition to its diagnostic capabilities, TMS also has therapeutic applications, particularly rTMS. rTMS is now approved for clinical use to treat patients with major depressive disorder and obsessive–compulsive disorder [21,22]. In the evidence-based guidelines published by Lefaucheur et al. [23], “Level A” (highest evidence rating) was reached for use of rTMS for neuropathic pain, depression, and hand motor recovery in the post-acute stage of stroke. Additional therapeutic rTMS protocols are currently under investigation after some promising results for migraine management, non-motor stroke recovery, various CNS pain syndromes, and post-stroke aphasia, using H-coil deep TMS [24]. However, many neurological applications remain underexplored or lack formal recommendations due to insufficient or inconsistent evidence from clinical trials, systematic reviews, or meta-analyses [25]. In this review, we discuss the dual role of TMS as both a diagnostic tool and a therapeutic modality, highlighting its ever-growing importance and versatility within the field of neurology. First, we summarize the outcome measures of TMS. Then, we examine these foundational techniques in the context of their diagnostic utility in selected neurological disorders, with a particular focus on PD, ET, dystonia, Alzheimer’s AD, and MCI. Lastly, we discuss how rTMS can be used to therapeutically modulate and potentially normalize cortical dysfunctions and the associated symptoms identified through diagnostic TMS. A secondary aim of this review is to emphasize the importance of standardized TMS protocols. By highlighting variability and inconsistencies in outcomes across studies, we underscore the need for rigorous and consistent methodology to ensure the reproducibility, comparability, and reliable interpretation of TMS findings.

## 2. Literature Search Methodology

**Figure 2:**
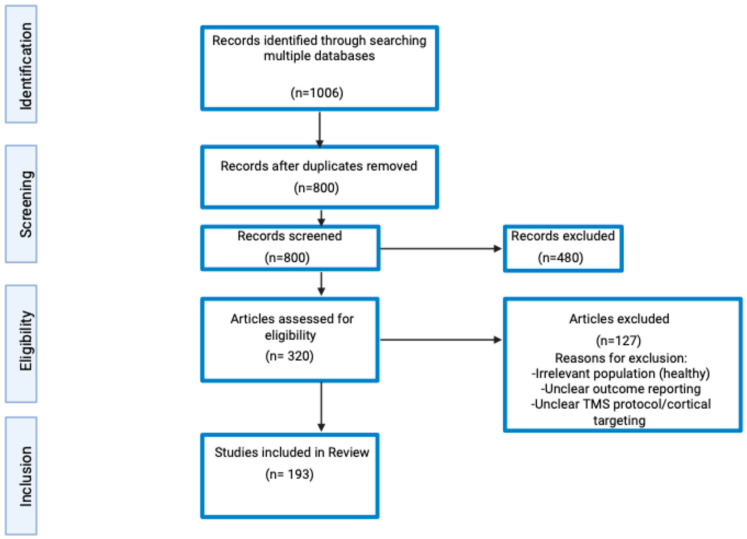
PRISMA-ScR flow diagram for the identification, screening, eligibility, and inclusion of studies on TMS outcome measures and rTMS protocols in movement and neurodegenerative disorders. Literature searches were conducted in MEDLINE, Google Scholar, and ClinicalTrials.gov to identify studies addressing the outcome measures and diagnostic or therapeutic utilities of TMS across neurological disorders. After the removal of duplicates, the titles and abstracts were screened, followed by full-text review to assess eligibility. Studies were excluded if they involved irrelevant populations, non-TMS interventions, or insufficient outcome reporting. A total of 193 studies were included in the final synthesis.

This review aimed to address the following question: “What are the outcome measures and the diagnostic and therapeutic utilities of TMS across various neurological diseases?”. A literature search following a scoping review framework was conducted using Google Scholar, MEDLINE, and clinicaltrials.gov databases, from 27 February 2025 to 28 December 2025. The following search terms were used in various combinations: “transcranial magnetic stimulation,” “outcome measures,” “motor thresholds,” “input–output curve,” “cortical silent period,” “short-interval intracortical inhibition,” “short-interval intracortical facilitation,” “long-interval intracortical inhibition,” “intracortical facilitation,” “interhemispheric inhibition,” “short-latency afferent inhibition,” “Parkinson’s disease,” “dystonia,” “essential tremor,” “Alzheimer’s disease,” “mild cognitive impairment,” “repetitive transcranial magnetic stimulation,” and “treatment.” Only English-language publications discussing outcome measures or the diagnostic and therapeutic applications of TMS in neurological conditions were included. The search results were screened to identify studies relevant to TMS outcome measures and rTMS applications in neurological disorders. Due to heterogeneity in reporting and study design, the review focuses on mapping key trends rather than exhaustive study enumeration. Refer to Figure 2.

## 3. TMS Outcome Measures

### 3.1. Measures of Corticospinal Tracts

#### 3.1.1. Motor Thresholds

MT is commonly defined as the minimum TMS intensity necessary to produce an MEP [6,11,26]. MT can be divided into the resting motor threshold (RMT) and active motor threshold (AMT). RMT typically refers to the lowest intensity necessary to evoke an MEP amplitude of ≥50 µV visually detectable on 50% of pulses [6]. Conversely, AMT is commonly defined as the minimum intensity necessary to evoke an MEP amplitude of ≥200 µV during slight isometric muscle contraction. RMT will always be higher than AMT because resting muscles require greater stimulation to produce a motor response, as the motor neurons are less excitable in the absence of voluntary activity. In contrast, during slight muscle contraction, neural excitability is heightened, allowing TMS to elicit responses at lower intensities [6].

MTs reflect the excitability of the core population of neurons in the M1 that are responsible for activating the target muscle, as well as the excitability of motor neurons in the brainstem or spinal cord. MTs can vary depending on factors such as the distance between the TMS coil and the underlying cortex, individual differences in cortical excitability, and even the specific muscle being stimulated [27]. Notably, MTs are lower in intrinsic hand muscles compared to those in the proximal arm, trunk, or lower limbs, underscoring the varying strength of corticospinal projections across different muscle groups.

#### 3.1.2. Input–Output Curve

The I/O curve illustrates the relationship between TMS intensity and the amplitude of the MEP, serving as a measure of corticospinal excitability. Steeper I/O curve slopes reflect greater neural responsiveness and are influenced by the degree of corticospinal neuron activation and the strength of their projections to the target muscle. Muscles with lower MTs, like intrinsic hand muscles, typically display steeper I/O curves [28].

It is important to note that MEP amplitudes can vary significantly even when the same TMS intensity is applied under consistent operator and subject conditions [29]. Some known physiological mechanisms underlying this variability include the fluctuation of neuronal excitability at corticospinal levels, the timing of the TMS stimulus application relative to the oscillatory states of the targeted neurons, and the activation of the target muscle [30]. To account for the inherent variability in MEP amplitudes, taking an average of multiple readings is essential in order to achieve reliable and accurate results. Relying on only a few measurements may lead to misleading conclusions due to the fluctuating factors that influence MEP response.

#### 3.1.3. Cortical Silent Period

The CSP is a temporary suppression of muscle activity, recorded as a quiet period in electromyography signals, that occurs after the onset of an MEP following TMS of the M1. It is a measure of intracortical inhibition, reflecting the inhibitory influence of the motor cortex on muscle activity [31]. The CSP duration is positively correlated with TMS intensity but is not influenced by the strength of the preceding MEP amplitude or the level of muscle contraction, indicating that CSP reflects intrinsic cortical inhibitory processes independent of peripheral variables [32,33,34]. Furthermore, the CSP can be elicited with subthreshold TMS intensity without a preceding MEP [35], further highlighting the reliability of the CSP to measure intracortical inhibition independent of confounding variables. The beginning half of the CSP is modulated by spinal inhibitory circuits, while the latter half is modulated by GABAergic transmission [34,36].

The ipsilateral silent period (iSP) is a measure of interhemispheric inhibition (IHI) between the two M1s, assessed by applying TMS to one hemisphere during voluntary muscle activity and observing a temporary suppression in the ipsilateral muscle [16]. This suppression reflects transcallosal inhibition, primarily mediated by the corpus callosum, though non-callosal pathways may also contribute [37]. The iSP provides insight into how effectively one hemisphere can inhibit motor activity in the other.

### 3.2. Measures of Intracortical Circuits Through Paired-Pulse Paradigms

#### 3.2.1. Short-Interval Intracortical Inhibition

SICI refers to the TMS paradigm used to evaluate motor cortex inhibition [38]. It involves delivering two magnetic pulses: a subthreshold conditioning stimulus (CS) followed shortly (usually 1–5 ms later) by a suprathreshold test stimulus (TS) over the same cortical area. The time difference between these two stimuli is termed the inter-stimulus interval (ISI). The result is a reduced motor MEP amplitude compared to when the test stimulus is delivered alone. SICI measures the strength of intracortical inhibition, and alterations in SICI can indicate dysfunction in inhibitory neurotransmission related to GABA_A_ receptors [39]. Through repeated testing, two maximum inhibition peaks have been identified, occurring at ISIs of 1 and 2.5–3 ms [40,41,42]. There are multiple methods of measuring SICI, including the “constant stimulus” [38] method, which applies a fixed subthreshold conditioning pulse followed by a suprathreshold test pulse to evaluate inhibition at a set intensity, and a threshold tracking paired-pulse paradigm [42], which adjusts the test stimulus intensity to maintain a constant MEP amplitude, offering a dynamic measure of inhibition. However, there seems to be no significant difference in reliability between them [43].

It is suggested that the first phase of SICI (0–2.5 ms) is mediated by the subthreshold CS recruitment of late oscillatory waves (specifically I3) from the TS, measured through epidural readings [44]. The second phase (2.5–3.5 ms) is suggested to be mediated through inhibitory inter-neuronal circuits acting via GABA_A_ receptors, as evidenced through pharmacological studies [45,46]. One study suggested that polymorphisms in brain-derived neurotrophic factor (BDNF) may affect the interhemispheric balance of SICI, indicating that inconsistencies in past SICI research could stem from individual differences in BDNF expression [47]. SICI does not seem to be influenced by gender [48], but does decrease with age [49], suggesting a reduced intracortical inhibitory function in older individuals.

Notably, SICI depends on the CS and TS intensities, because it is absent if TS is <110% of RMT [50], and increases with greater TS intensities [51]. Moreover, SICI is weak or absent at low CS intensities, increases as CS intensity rises, but then diminishes again, and may even become facilitatory when the CS approaches the RMT, resulting in a U-shaped response curve [28,52]. Additionally, SICI is influenced by both the contraction state of the muscle and the TMS coil type [53,54,55]. Overall, SICI may help refine motor cortex output by promoting the selective activation of target muscles while suppressing the unintended activation of other muscles.

#### 3.2.2. Short-Interval Intracortical Facilitation

SICF is a paired-pulse TMS protocol that assesses excitatory circuits within the M1, likely reflecting activity of corticospinal neurons and their facilitatory interneurons [56,57]. It involves delivering two suprathreshold pulses (S1 and S2) at specific ISIs (typically peaking at 1.1–1.5 ms, 2.3–2.9 ms, and 4.1–4.4 ms), which produce enhanced MEPs compared to TS alone [58,59,60]. SICF is used to measure intracortical excitability and temporal summation of excitatory inputs, providing insight into the timing and function of I-waves in corticospinal output [28].

Despite the precise physiological mechanisms underlying SICF having yet to be fully explained, it has been proposed that the facilitatory interactions of oscillatory I-waves form the basis of SICF [46,56,59]. Specifically, TMS modeling studies suggest that suprathreshold S1 leads to subliminal depolarization of cortical neurons [61]. A subsequent S2 pulse applied within a short ISI causes the cortical neurons to reach the action potential threshold, generating an MEP [61,62,63]. Further support for a cortical origin is demonstrated in pharmacological studies, which suggest a variety of neurotransmitter-mediated effects on SICF [46,60,64]. Furthermore, the stimulation intensities and ISIs used for SICI can overlap with those that recruit SICF, potentially explaining the observed reduction in SICI at higher CS intensities. To minimize this confounding effect, guidelines recommend keeping the CS intensity below AMT and selecting ISIs that correspond to SICF troughs. This approach not only reduces SICF’s influence on SICI but also reinforces the notion that SICF originates and is mediated within the cortex [6,65]. Lastly, voluntary muscle contraction and age do not seem to affect SICF [16]. Contrarily, coil type does seem to affect SICF, with the figure-of-eight coil increasing SICF [53,66].

#### 3.2.3. Long-Interval Intracortical Inhibition

LICI is a paired-pulse TMS protocol that measures inhibitory intracortical circuits. It involves delivering an S1 followed by an S2 with an ISI of 50–200 ms [42]. LICI has well-established cortical origins, with studies revealing epidural recordings indicating reduction in corticospinal test volleys [67,68]. It has been suggested that LICI is mediated through GABA_B_ receptors and may be increased through the introduction of GABA_B_ agonists, analogs, and uptake inhibitors [69]. Evidence shows that LICI decreases with increasing TS (S2) intensities [70], but is not affected by muscle contraction [71]. Triple-pulse TMS studies show that LICI can inhibit SICI, providing further evidence that both are mediated through GABA_B_ pathways [72].

#### 3.2.4. Intracortical Facilitation

ICF is a paired-pulse TMS protocol that measures excitatory cortical circuits by applying a subthreshold CS followed by a suprathreshold TS at ISIs of 8–30 ms, with the most prominent facilitation seen at 10–15 ms [42]. ICF leads to an increase in MEP amplitude, which reflects excitatory glutamatergic and serotonergic activity, likely mediated by NMDA receptors. This is demonstrated by the reduction in ICF following the administration of NMDA antagonists like dextromethorphan and the increase in ICF following the administration of SSRIs [73]. Evidence supporting a cortical origin for ICF includes the fact that it can be elicited using subthreshold CS, which does not generate descending corticospinal activity [74]. Additionally, epidural recordings show no changes in the amplitude or number of corticospinal volleys during ICF, further indicating that the effect arises from intracortical mechanisms rather than spinal contributions [74].

#### 3.2.5. Interhemispheric Inhibition

IHI refers to the process by which one motor cortex suppresses activity in the opposite motor cortex via transcallosal pathways [75]. It plays a critical role in coordinating bimanual movements and maintaining motor control by preventing unwanted mirror activity. IHI can be evoked through the paired-pulse stimulation of a suprathreshold CS to the M1 followed by a suprathreshold TS to the opposite M1 [75,76]. Two types of IHI have been documented: short interval interhemispheric inhibition (SIHI) between 6 and 11 ms (peak at ~9.6), and long-interval interhemispheric inhibition (LIHI) between 20 and 50 ms [77]. IHI occurs at the cortical level, as evidenced by epidural readings showing an association with the reduction in oscillatory waves (particularly I_3_) [78]. The proposed mechanism involves the conditioning stimulus activating excitatory transcallosal fibers, which in turn exert an inhibitory effect on the opposite M1 by engaging inhibitory GABAergic receptors [75,79]. For the purposes of this review, the distinction between SIHI and LIHI is not relevant in later sections, but the interested reader is encouraged to consult further detail on SIHI and LIHI [63,72,75,80].

#### 3.2.6. Short-Latency Afferent Inhibition

Short-latency afferent inhibition (SAI) assesses sensorimotor integration by pairing peripheral nerve stimulation with TMS over M1, resulting in a reduction in MEP amplitude [81]. It is known that afferent input from hand innervation may cause cortical inhibition if delivered 18–28 ms prior to TMS of M1 [72]. The ISI depends on the target muscle, with digital nerves having longer ISIs than more proximal hand muscles [82]. Consequently, the peak ISIs vary by muscle [6]. However, SAI reaches its maximal level at an intensity that recruits all sensory afferent action potentials [83,84]. SAI occurs at the cortical level as evidenced by epidural readings showing the inhibition of oscillatory I-waves appearing unrelated to spinal cord changes [85,86]. SAI is mediated by cholinergic pathways and is reduced by GABAergic pathways [87,88]. Lastly, SAI does not seem to be affected by age or gender [89].

#### 3.2.7. Long-Latency Afferent Inhibition

Long-latency afferent inhibition (LAI), like SAI, evaluates sensorimotor integration by combining peripheral nerve stimulation with TMS applied over M1. The primary distinction lies in the ISI, which, for LAI, typically ranges from ~200 to 1000 ms and produces a reduction in subsequent MEP amplitude [90]. Similarly to SAI, the optimal ISI varies depending on the muscle targeted; however, due to the longer interval between peripheral and cortical stimulation, the afferent signal is thought to be transmitted mainly via GABAergic rather than cholinergic pathways [91,92]. Accordingly, LAI is mediated primarily by GABA_A_ receptor circuits [93]. Importantly, LAI appears to also be unaffected by age or gender [89].

#### 3.2.8. Cerebellar Inhibition

Cerebellum-to-motor cortex inhibition (CBI), refers to the modulatory effects of cerebellar stimulation on the contralateral motor cortex [94]. The most reliable way to induce CBI is by using a double cone coil, positioned over the midpoint on a line between the inion and eternal auditory meatus, typically about 3–5 cm lateral to and 0–2 cm above the inion [95]. For standard double-cone stimulation, the CS is usually delivered at an intensity 5–10% below the AMT [96]. Because activating deep Purkinje cells requires relatively strong stimulation, the CS intensity is often high and can be uncomfortable for participants. Despite this drawback, the double-cone coil produces the most consistent CBI effects, and increasing the CS intensity beyond 60% of the maximum stimulator output does not produce further inhibition [95]. Importantly, a CBI-like reduction does not occur when the TS is delivered directly over M1, which indicates that the mechanisms responsible for CBI happen at the cortical level. CBI produces suppression of the MEP at ISIs of 5–8 ms, which reflects cerebellar activation and spinal inhibitory mechanisms, particularly around ISIs of 7–8 ms [95,97]. More specifically, Purkinje cells in cerebellar lobules V–VIII are responsible for mediating this inhibition [98]. Their activation decreases the tonic facilitatory output of the dentate nucleus onto the contralateral M1 through the dentate–thalamo–cortical pathway [99]. It is also important to recognize that two independent cerebello-M1 pathways may contribute to CBI, and both should be considered in pathophysiological investigations [100]. One pathway is probed using posterior-to-anterior currents, which influence the excitability of layer V pyramidal neurons in M1, while the other is assessed with anterior-to-posterior currents and targets neurons in the premotor cortex that project to M1 [100].

## 4. Diagnostic Utility of TMS Abnormalities in Neurological Diseases (Table 1)

Before beginning this section, it is important to note that while many TMS outcome measures have demonstrated reproducible results, some findings vary across studies, reflecting differences in stimulation parameters, cortical targets, patient populations (including medication status), and study designs. The scope of this review is to acknowledge and highlight these inconsistencies to reiterate the need for protocol standardization.

**Table 1:** Consensus on the diagnostic utility of TMS in neurological disorders.

Condition	Unique TMS Outcomes	Prospective Value
Parkinson’s Disease (PD)	SICI ↓ ⟷ * CSP duration ⟷ ↓ LICI (⟷ ↓ ↑) * SICF ↑ ICF ⟷ ↓ AMT⟷ ↓ (Symptomatic influence) RMT ⟷ ↓ (Symtpomatic influence) I/O ↑ (Steeper curve at rest) SAI ↓ (disease progression + dopaminergic therapy) ⟷ ↑ (in patients not receiving dopaminergic therapy) CSP duration ⟷ ↓ ISP duration ⟷ ↓ LIHI ↓ SIHI ⟷ ↓	SAI may help predict the development of Parkinson’s disease-related dementia and the risk of falls. Potential for SICI and SICF to serve as biomarkers for tracking disease progression.
Dystonia	RMT ⟷ I/O ⟷ SICI ↓ ⟷ * LICI ↓ (rest) ⟷ ↑ ↓ (active) * ICF ↓ (sometimes absent) CSP ↓ SIHI, LIHI ↓ (with mirror movements + severity dependent) LAI, SAI ↓ ⟷ (subtype dependent) Surround inhibition ↓ CBI ↓ ⟷ (subtype dependent + severity dependent)	Diagnostic value remains limited, and further research is needed to clarify differences across subtypes.
Essential Tremor (ET)	CBI (↓ ⟷) * SICI (↓ ⟷) * SAI ↑ (time dependent) LICI ⟷ ↓ (stimulation dependent) RMT (↑ ↓, drug dependent) * AMT ↓ * I/O (⟷ ↓) * CSP ⟷	Diagnostic value remains limited, and additional research is needed, particularly on drug interactions.
Alzheimer’s Disease (AD)	RMT, AMT ↓ (severity dependant) CSP duration ⟷ SICI (⟷) ↓ (time dependent) * ICF ↓ LICI (↓ ⟷) * CSP ⟷ SAI ↓ (normalized with dopaminergic and cholinergic therapies)	TMS-based profiles using multiple outcome measures can distinguish AD from other neurodegenerative disorders with up to 92% accuracy.
Mild Cognitive Impairment (MCI)	RMT, AMT ↓ (apparent, but statistically non-significant) SICI ⟷ ICF ⟷ LICI ⟷ SAI ↓ (subtype dependent)	The SICI-ICF/SAI ratio has been shown to differentiate AD-related MCI from non-AD MCI with 90% accuracy, performing comparably to amyloid biomarkers.

AMT: Active motor threshold, CBI: cerebellar inhibition of the motor cortex, CSP: cortical silent period, I/O: input–output curve, ICF: intracortical facilitation, ISP: ipsilateral silent period, LAI: long-latency afferent inhibition, LICI: long-interval intracortical inhibition, LIHI: long-interval interhemispheric inhibition, RMT: resting motor threshold, SAI: short-latency afferent inhibition, SICF: short-interval intracortical facilitation, SICI: short-interval intracortical inhibition, SIHI: short-interval interhemispheric inhibition, ⟷, no change or normal, ↓: reduced, ↑: increased, and * not general consensus, but reported in some discordant findings.

### 4.1. Parkinson’s Disease

PD is a neurodegenerative disorder that has been classically characterized primarily by the loss of dopaminergic neurons, particularly in the substantia nigra [101]. More recently, however, a new classification system, the SynNeurGe research diagnostic criteria, was proposed, which emphasizes the biological markers associated with PD [102,103]. This new system classifies PD based on the presence or absence of the pathological α-synuclein “Syn” in the cerebrospinal fluid or peripheral tissue, neuroimaging features defining the presence of neurodegeneration “Neur”, and the presence of Parkinson’s disease-specific pathogenic gene variants “Ge” [103]. Regardless of classification, PD affects 1% of the population aged over 65 years, and is clinically characterized by muscle rigidity (stiffness), tremors (shaking) at rest, bradykinesia (slowness of movement), and postural gait disorders (balance impairment), although the exact cause of PD remains unclear [103].

In PD patients, reduced SICI [65,104,105,106] and CSP duration [107,108] have been reported, indicating the dysfunction of the GABAergic pathways. Despite a reduced SICI being the consensus in PD, there have been reports of unaffected SICI [109,110]. Notably, MacKinnon et al. [110] used a CS intensity of less than 80% of the RMT and observed no significant effect on SICI. However, when they increased the CS to greater than 80% of RMT, they reported reduced SICI. This pattern suggests that the low-threshold inhibitory circuits mediating SICI remain intact in PD, while the suppression observed at higher CS intensities may reflect a lower threshold of intracortical facilitatory circuits, which could interfere with inhibitory processes in PD. Furthermore, Hanajima et al. [109] found that when using anterior–posterior-directed currents, SICI remained unchanged, whereas with posterior–anterior-directed currents, SICI was reduced. Multiple studies suggest that the increased inhibition seen is consistent across all PD patients, regardless of medication status or the presence of dyskinesia [111]. Moreover, as PD progresses, there is continued reduction in SICI [112]. Some studies have suggested that dopaminergic medications normalize SICI [65,104], while others have not seen such effects [110,113]. While this may be due to variability within PD patients, a recent cross-sectional study has suggested that cortical disinhibition, measured through SICI, is largely dopamine independent [114]. Lastly, the reduction in SICI is more pronounced on the diseased side, including in levodopa-naïve patients [111]. Conversely, the LICI function in PD is more ambiguous, with some studies reporting an increase [108,115], a decrease [116], or no change in LICI [91]. This variability may stem from differences in disease stages, medication status, and methodological approaches.

AMT and RMT were found to be generally unaffected in PD patients [117,118,119], regardless of medication state [113,119,120]. However, some studies do report decreased AMT and RMT [121,122,123,124], although this is most likely due to bradykinetic symptoms influencing the voluntary contractions of the participants. Furthermore, both MEP amplitudes and I/O curve steepness were found to be increased at rest and decreased during muscle activity [113,123], indicating a heightened corticospinal excitability, most likely a compensatory mechanism from bradykinetic symptoms.

Studies demonstrate that ICF is reduced [125,126,127] or normal [104] in PD. However, SICF specifically is increased, and this increase is associated with a reduced SICI [65,128], potentially indicating a causal relationship. The increase in SICF was observed in both levodopa-naïve patients [129] and seems to increase in dyskinetic PD patients [130]. Interestingly, one study found that when SICI and SICF-1 (ISI 1.1–1.5) were assessed together, the results were not significantly different from healthy controls, suggesting that altered cortical interactions may be an aspect of PD and could be linked to disease progression [129].

LIHI was found to be reduced in PD patients with mirror movements despite no change in SIHI [131], suggesting dysfunction of the corpus callosum and interference in interhemispheric communication. Further investigation found that both SIHI and LIHI were reduced compared to healthy controls, with no significant hemispheric difference [119]. When analyzing repetitive finger movements, an imbalance in SIHI (less inhibition from the less to the more affected M1) was linked to the sequence effect in the most affected hand [119]. They also observed a positive correlation between PD SIHI imbalance and MDS-UPDRS-III scores, suggesting that IHI imbalance may contribute to bradykinesia features in PD. Importantly, while dopaminergic therapy did not restore the impaired IHI or the sequence effect as measured kinematically, it did influence the relationship between SIHI imbalance and bradykinesia in PD patients [119].

SAI has been found to be either normal or enhanced in patients not receiving dopaminergic therapy [132], but appears reduced in those on dopamine medications [91]. SAI in medicated patients has been positively correlated with improved gait parameters, such as increased gait speed and step length, and was shown to partially account for the variability in gait speed [133]. Additionally, lower SAI levels have been observed in PD patients with a history of falls, even after controlling for cognitive status, suggesting its potential as a predictive biomarker for gait, balance, and postural instability [134]. Reduced SAI has also been linked to cognitive decline in PD [135,136,137]. Conversely, there is evidence suggesting no change in SAI between PD populations and controls [138]. One possible explanation is that the over-inhibition of non-cholinergic neurons within the pedunculopontine nucleus [139], combined with relative preservation of cholinergic neurons [140], may lead to sustained or even enhanced cholinergic output. This mechanism could obscure underlying dysfunction and account for the observation of normal or elevated SAI levels in some patients.

### 4.2. Dystonia

Dystonia is characterized by involuntary, sustained muscle contractions involving both agonist and antagonist muscles, resulting in abnormal postures, twisting, repetitive movements, or tremors, which can be triggered or aggravated by voluntary movements [141]. Dystonia affects roughly 0.02% of the population [142], making it the second most common movement disorder after tremor. Dystonia varies in severity depending on activity, posture, and type [141]. The pathophysiology of dystonia includes the impairment of sensorimotor integration, a partial loss of the inhibitory control of the CNS, and changes in synaptic plasticity [143]. Collectively, these mechanistic abnormalities contribute to an impairment of the basal ganglia, resulting in an insufficient inhibition of noisy activity and an excessive excitation of cortical areas [143]. Additionally, beyond the traditional focus on the basal ganglia, growing evidence from animal, genetic, imaging, and electrophysiological studies implicates the cerebellum in dystonia. Specifically, the cerebello-thalamalo-cortical (CTC) pathway and its interaction with basal ganglia circuits appear to play key roles in the disorder’s pathophysiology [143].

Although RMT and IO recruitment curves are generally within normative ranges [144,145,146,147,148,149], several investigations have consistently revealed compromised intracortical inhibition mechanisms, including reductions in SICI, CSP, and a diminished or absent ICF response [104,150,151,152,153]. Furthermore, reductions in SICI have been reported across multiple dystonia subtypes, including idiopathic dystonia [154], dopa-responsive dystonia [155], and even asymptomatic carriers of DYT-1 gene mutations [156], suggesting disrupted cortical inhibitory interneuron activity. Nevertheless, several studies have reported conflicting results, demonstrating normal SICI responses in cervical and focal hand dystonias [157,158,159,160,161] as well as in muscle tension dystonia [162]. These discrepancies do not appear to stem from differences in the dystonia phenotype or somatotopic representation, as reduced SICI has also been recorded from unaffected limbs in cervical dystonia [163]. Several factors may underlie these conflicting results; therapeutic intervention with botulinum toxin, for example, has been shown to normalize SICI approximately one month post injection [164]. Methodological variability and inter-individual differences are also likely contributors [165].

LICI displays similar variability. In patients with writer’s cramp, LICI at rest is often reduced [150], while active LICI has mixed findings, having been reported as normal [150,161,166], diminished [71], or increased [160]. Similarly, CSP findings are also discordant, with some studies demonstrating shortening observed during fine motor tasks, but not during maximal voluntary contractions [167,168,169], and with evidence suggesting that premotor conditioning may further decrease CSP [170]. Furthermore, unlike with SICI, botulinum toxin does not seem to correct shortened CSP duration in dystonia [171]. Additionally, in writer’s cramp, iSP prolongation has been reported, potentially reflecting hyperactivation of transcallosal inhibitory pathways [172]. Interhemispheric inhibitory dynamics, including SIHI and LIHI, further delineate neurophysiological heterogeneity. Focal hand dystonia patients often exhibit reduced SIHI and LIHI on the affected side, particularly at movement initiation and in the presence of mirror dystonia [173,174]. In more severe phenotypes, bilateral reduction in IHI has been observed [175]. Notably, in musician’s dystonia, these impairments extend to unaffected first-degree relatives, supporting the role of interhemispheric dysfunction as a potential biomarker [176].

Sensory–motor integration is also disrupted. LAI, which is normally inhibitory at an ISI of 200 ms, is surprisingly facilitatory in focal hand dystonia, particularly at the onset of movement [177]. However, this reversal is not observed in cervical dystonia, suggesting subtype-specific pathophysiology. These findings indicate that impaired LAI alone cannot fully account for reduced surround inhibition or dystonic contractions [178]. SAI, which reflects sensory–motor integration mediated by cholinergic and GABAergic mechanisms, also shows regionally restricted deficits. For example, focal hand dystonia patients demonstrate topographically limited reductions in SAI when stimulated via digital nerves [152], whereas median nerve stimulation may elicit facilitation at ISIs such as 20–30 ms, but normal responses at longer intervals [145,179]. One investigation reported an unexpected enhancement of SAI in individuals with focal hand dystonia [180]. This paradoxical finding may be attributable to a reduction in surround inhibition during voluntary movement, likely linked to decreased GABAergic neurotransmission, which could account for the elevated digit-specific SAI observed.

Cerebellar involvement in dystonia has been increasingly recognized. Focal hand dystonia is associated with reduced CBI, pointing to dysfunction along the cerebello-thalamo-basal ganglia axis [153,181]. This reduction, however, is not observed in cervical dystonia [182], suggesting mechanistic divergence between the phenotypes. Furthermore, the cerebellar modulation of cortical circuits, typically observed as enhanced ICF and suppressed SICI in healthy individuals, is notably absent in dystonia [153]. The degree of CBI reduction correlates with cervical dystonia severity, supporting a contributory role of CTC pathways in focal dystonias [183].

### 4.3. Essential Tremor

ET is an adult movement disorder that exists in approximately 0.9% of the general population [184,185]. It is characterized by a postural kinetic tremor typically involving the upper limbs, along with the head, lower limbs, or voice. ET is widely recognized as a centrally driven tremor that does not arise from any independent structure, but rather results from dysfunction within the CTC network that governs motor control [186]. The cerebellum, particularly its Purkinje cells, plays a central role, with abnormal oscillatory activity and impaired inhibitory output contributing to tremor generation. This abnormal activity is relayed via the ventral intermediate nucleus of the thalamus to the M1, where it results in rhythmic muscle contractions [186]. The inferior olive may act as a pacemaker, further promoting tremor-related oscillations within the loop. Disruptions in this circuit, including increased synchronization and reduced inhibition, underlie the pathophysiology of ET [186].

CBI was reported to be reduced in ET but did not correlate with tremor severity [187]. This suggests that cerebellar efferent pathway dysfunction may reflect either a primary pathological process or a compensatory response to extracerebellar disease activity but is unlikely to fully account for the pathophysiology of ET. However, there have also been reports of normal CBI in ET [188]. A recent study further investigated the role of CTC connectivity in ET by assessing CBI in patients who underwent focused ultrasound thalamotomy [114]. The results showed that CBI was reduced following thalamotomy, supporting the involvement of the CTC pathway in ET though no significant difference in CBI was found between ET patients and healthy controls. Notably patients exhibited reduced SICI, suggesting potential compensatory interactions between cerebellar and cortical circuits which was further supported by other studies [189,190]. Contrarily, early literature revealed conflicting results showing no changes in SICI between ET and healthy populations [191,192]. SAI has been reported to increase in ET compared to healthy controls and appears to be time dependent, with higher SAI at ISI 22 ms than at ISI 24 ms [190]. LICI was reported to be normal in ET [191,193], but significantly reduced in paired associative stimulation [193]. Interestingly, in this regard, ET behaves more similarly to healthy controls than to other tremor patients, including dystonic and primary writing tremor patients [193]. Furthermore, CSP has also been reported to be normal in ET [191,194].

ET patients show higher RMT but no differences in the I/O curve compared to healthy controls, indicating reduced M1 excitability [190]. However, these findings are discordant, as there are reports of reduced AMT, RMT, and I/O curves in ET [195] and reports of no significant change [196]. A recent pharmacological study has further clarified this variability; primidone treatment led to an increase in RMT, likely due to its effect on voltage-gated sodium channels. Additionally, it reduced both the resting and active I/O curves, prolonged the CSP, enhanced LICI and SAI, and reduced SICI, indicating the modulation of both GABA_B_- and GABA_A_-mediated neurotransmission. In contrast, propranolol’s central effects were more limited—primarily reducing the resting I/O curve and enhancing SAI—implying that its mechanism may involve noradrenergic regulation of GABAergic activity [197].

### 4.4. Alzheimer’s Diseases and Mild Cognitive Impairment

AD, named after the German psychiatrist Alois Alzheimer, is the most prevalent form of dementia. It is a progressive neurodegenerative disorder marked by the accumulation of amyloid beta peptide (Aβ), leading to the formation of neuritic plaques and neurofibrillary tangles [198]. These pathological changes primarily affect the medial temporal lobe and neocortical regions of the brain, impacting memory and cognition.

AD is associated with increased motor cortex excitability, as demonstrated by reductions in both RMT [199,200,201,202,203,204,205,206,207] and AMT [202,208,209] which significantly correlates with disease severity [210]. This hyperexcitability reflects altered cortical neurotransmission dynamics in the M1, likely stemming from disrupted interactions among GABAergic, glutamatergic, and cholinergic systems, ultimately resulting in an excitatory–inhibitory imbalance [199]. Since TMS induces rapid, repetitive firing of pyramidal neurons, an activity pattern primarily mediated by non-NMDA glutamatergic receptors, it has been suggested that the heightened cortical excitability observed in AD may be driven by increased signaling through these non-NMDA receptors [199,211].

Studies have reported discordant results regarding SICI. While some suggest significant SICI reductions [212,213,214,215], others have reported SICI as unaltered in AD [200,204,216,217,218,219,220]. Two recent meta-analyses were conducted, with one indicating that SICI impairment may emerge predominantly in individuals with prolonged disease duration [221], suggesting that discrepancies among studies could be attributable to heterogeneity in patient populations and disease staging. The other suggests the overall reduction in SICI in AD, also correlated with disease severity and associated with GABA_A_-mediated inhibition [222]. Similarly, LICI has been shown to be reduced in several reports [211,218]. Conversely, most investigations report normal CSP duration in AD [201,215,223,224,225,226]. Although findings on GABA_A_- and GABA_B_-mediated pathways in Alzheimer’s disease are mixed, the emerging evidence highlighting their role remains promising, underscoring the need for further research to clarify their specific contributions to the disorder.

ICF has been reported to be decreased in AD [200,204,215,216,217,218,220,227,228]. Moreover, SAI, which is a cholinergic-mediated process, is consistently found to be markedly reduced in AD patients [204,207,218,227,229,230,231,232,233,234]. Notably, pharmacologic interventions including acetylcholinesterase inhibitors [199], L-DOPA [203,234] and dopamine agonists [217] have been shown to normalize SAI responses. Alterations in interhemispheric connectivity have also been reported in AD, with prolonged iSP latencies indicating impaired transcallosal inhibition [208,213].

MCI is an intermediary condition between normal aging and dementia [235]. Roughly 28.7% of individuals with MCI progressed to dementia over a five-year follow-up, with a linearly increasing cumulative risk each year (from 5.4% to 42.5%) [236]. Several studies have reported a non-significant increase in M1 excitability in MCI, resembling that observed in AD [237,238,239,240,241]. However, SICI and ICF did not significantly differ in MCI compared to controls across most studies [237,238,239,241], while SAI was frequently reduced [227,237,239,241,242], with the variability in findings depending on the MCI subtype [238,240]. Notably, long-term potentiation (LTP)-like plasticity appeared to remain preserved in MCI [243].

TMS has shown strong potential in differentiating AD from other neurodegenerative disorders [17]. While individual TMS metrics like MTs lack disease specificity, combining multiple measures such as SICI, ICF, SAI, and LICI significantly improves diagnostic accuracy [218]. Large-scale studies, including one utilizing machine learning, have demonstrated that TMS-based profiles can distinguish AD with up to 92% accuracy, underscoring its diagnostic utility [227]. The diagnostic utility of TMS in terms of differentiating MCI subtypes has also been demonstrated, with one single-center study reporting up to 90% accuracy in distinguishing AD-related from non-AD-related MCI using a composite index ([SICI–ICF]/SAI), achieving 87.9% specificity and 94.4% sensitivity, comparable to amyloid biomarkers [239,244]. A larger investigation further validated these findings using a machine learning algorithm incorporating SICI, ICF, SAI, and LICI, yielding diagnostic accuracies between 72 and 86%, with precision and recall ranging from 72 to 90% and 75–98%, respectively [237].

## 5. rTMS in the Treatment of Neurological Disorders (Table 2)

**Figure 3:**
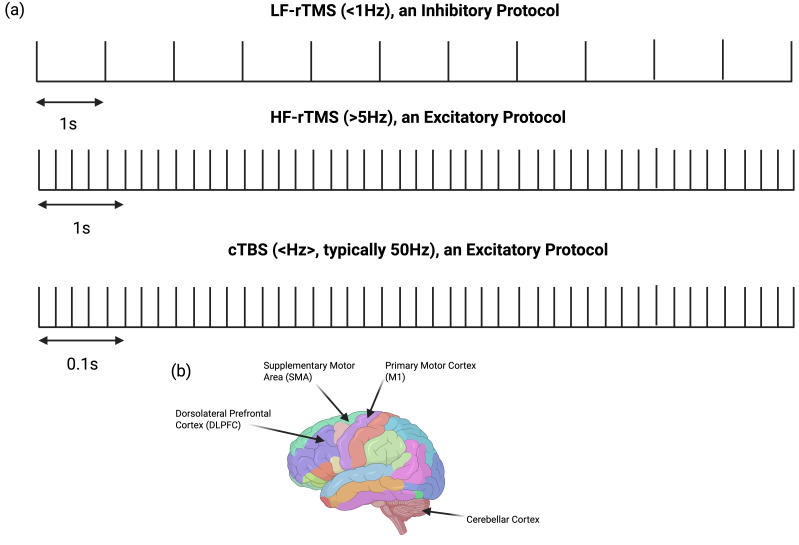
Overview of rTMS stimulation frequencies and commonly targeted cortical regions. (**a**) Schematic representation of commonly used low- and high-frequency rTMS protocols and their typical excitatory or inhibitory effects, alongside (**b**) the cortical regions frequently targeted in clinical and research applications. Highlighted regions reflect common stimulation sites across neurological and neuropsychiatric disorders, including the primary motor cortex, supplementary motor area, dorsolateral prefrontal cortex, and cerebellar cortex, all relevant targets within this section.

Similarly to Section 4, it is important to note that while rTMS has shown therapeutic benefits for movement disorders, its efficacy varies across studies. Again, these differences likely reflect variations in stimulation parameters, cortical targets, patient populations, and study designs. The scope of this review is to acknowledge and highlight these inconsistencies, emphasizing the need for standardized protocols to optimize clinical outcomes. Readers interested in a quantitative synthesis are encouraged to consult other systematic reviews that report pooled efficacy estimates for various movement disorders and their specific symptoms. An overview of rTMS stimulation frequencies and commonly targeted cortical regions is provided in Figure 3.

### 5.1. Parkinson’s Disease

While a cure is not yet available, current treatment strategies for PD target symptom relief and primarily involve medications such as levodopa–carbidopa, dopamine agonists like ropinirole and pramipexole, and monoamine oxidase B inhibitors such as selegiline and rasagiline [245]. Adjunct therapies, including physical therapy, are proposed to slow the progression of the disease [246,247]. Unfortunately, for individuals who experience significant side effects or fail to respond to medication, there are limited options, with deep brain stimulation, an invasive surgery with its own risks and complications [248], being the most promising in select patient populations [246,249,250]. This highlights the need for further research into non-invasive treatments that can alleviate symptoms.

rTMS has emerged as a promising therapeutic intervention for PD, with effects spanning both motor and non-motor symptoms [23,251]. Its mechanisms appear to involve changes in neurotransmitter release, transsynaptic efficiency, signal transduction, gene transcription, and neuroplasticity [252,253,254,255,256]. Research suggests that rTMS promotes neurogenesis, neuronal survival, and the release of neuroprotective compounds in PD patients [252,257,258]. One proposed mechanism is that high-frequency rTMS (HF-rTMS), an excitatory protocol, enhances activity in the caudate nucleus and helps restore dopamine balance within the nigrostriatal-thalamo-cortical pathway. [259]. For instance, stimulation over the M1 has been shown to influence dopamine release in nigrostriatal regions [23,260,261]. Building upon these mechanistic insights, various rTMS protocols have been developed and investigated for the treatment of PD, differing primarily in stimulation frequency—high versus low—and in the selection of cortical targets, each tailored to address specific motor and non-motor symptoms.

#### 5.1.1. High-Frequency (>5 Hz) rTMS for Motor Symptoms

Numerous studies have evaluated the effects of 5 Hz rTMS over M1. One study [262] demonstrated that subthreshold 5 Hz rTMS led to short-term improvements in motor function, particularly in the upper limb contralateral to the stimulation site and mild ipsilateral improvements possibly via transcallosal modulation. These changes are attributed to enhanced basal ganglia–thalamocortical outflow, reinforcing motor loop activity. Long-term benefits of 5 Hz rTMS were also observed in another study [263], potentially due to increased dopamine release in the striatum and prefrontal cortex. The stimulation of corticostriatal axons in the prefrontal cortex may lead to endogenous dopamine release in the caudate nucleus [264], suggesting that intracortical modulation may underlie the beneficial effect [263].

In addition to M1 stimulation, various rTMS targets have been proposed for alleviating specific PD symptoms. HF-rTMS over M1, broader motor cortex regions (e.g., limb specific), and the dorsolateral prefrontal cortex (DLPFC) has been linked to improvements in both motor and mood symptoms [265,266]. Lefaucheur et al. [23] emphasized that although M1 stimulation with HF-rTMS consistently improves motor symptoms, its clinical application still demands stronger evidence from longitudinal trials. Notably, rTMS has also demonstrated efficacy in reducing levodopa-induced dyskinesia (LID) when applied to M1, DLPFC, and the supplementary motor area (SMA) [267,268], though the durability of these effects remains uncertain. Furthermore, the stimulation of the left DLPFC has been associated with improvements in depressive symptoms in PD; however, conflicting results from two randomized controlled trials [269,270] highlight the need for continued investigation in this area.

Further studies have explored stimulation of the SMA. One study [271] found that multi-week 5 Hz rTMS over the SMA enhanced activity in underactive neurons, likely contributing to the restoration of the basal ganglia–thalamocortical circuitry and motor function. Interestingly, another study reported that 1 Hz, but not 10 Hz, stimulation over the SMA improved motor symptoms in PD. However, these improvements were transient and dissipated during the observation period. [272]. Additionally, it was found that 10 Hz stimulation of M1 increased ICF and prolonged CSP, suggesting the restoration of both excitatory and inhibitory circuits [127]. Interestingly, while 0.5 Hz rTMS improved bilateral rigidity and gait via increased inhibitory drive (as evidenced by longer CSP and increased ICI), 1 Hz stimulation modulated excitability through mechanisms distinct from those in healthy controls [273,274,275,276]. Similarly, 10 Hz rTMS applied over M1 has been shown to reduce bradykinesia and improve rigidity, with contralateral specificity [127].

Several recent studies have investigated 10 Hz rTMS for freezing of gait (FOG), particularly in ON-OFF FOG patients. Two studies [277,278] reported that 10 Hz rTMS over the M1 region controlling the lower leg on the dominant hemisphere (M1-LL) significantly improved FOG. Mechanistically, increased cortical excitability and striatal activation may modulate globus pallidus interna inhibition [279]. Another study [280] also reported that 10 Hz rTMS over M1 improved SICI at the abductor pollicis brevis, suggesting cortical disinhibition plays a role in FOG and may be reversed by rTMS. It also demonstrated an improvement in gait speed and stride length, and an improved variability of gait speed. These findings highlight SICI as a potential biomarker for both FOG severity and treatment response, especially when measured at the 4-millisecond mark from the TS.

Moreover, 25 Hz rTMS has shown promising results. One study [281] observed cumulative improvements in gait and bradykinesia immediately and one month post treatment. Although repeated sessions showed reduced MEP amplitude (suggesting habituation or adaptive neural responses), patients with less severe baseline bradykinesia exhibited greater motor improvements. These findings imply that baseline symptom severity may influence responsiveness. Mechanistically, benefits may arise from dopamine release, NMDA receptor upregulation [282], or synaptic remodeling [283]. Neuroimaging studies confirmed rTMS-induced changes in functional connectivity between prefrontal and SMA regions, lasting up to 3 months, further supporting its role in motor improvement [259,284].

#### 5.1.2. Low-Frequency (<1 Hz) rTMS for Motor Symptoms

A meta-analysis of randomized controlled trials by Zhu et al. [285] was conducted to assess the efficacy of low-frequency rTMS (LF-rTMS), an inhibitory protocol, in improving the motor symptoms of PD. It identified eight trials involving a total of 319 patients. The analysis revealed that LF-rTMS applied to various structures including SMA, right- and left-DLPFC, and M1 regions (0.2 ≤ x ≤ 1 Hz) led to a significant reduction in motor symptoms, as indicated by a decrease in the Unified Parkinson’s Disease Rating Scale part III (UPDRS-III) scores, with an effect size of −0.40 (*p* < 0.05). The findings suggest that LF-rTMS may be a beneficial treatment option for managing motor symptoms in PD, potentially being more acceptable than HF-rTMS due to fewer side effects. However, the authors noted the limited number of included trials and patients, recommending further large-scale multi-center randomized controlled trials to validate their conclusions and explore optimal stimulation parameters. Overall, this study highlights the potential of LF-rTMS as a promising adjunctive therapy for PD motor symptoms.

Since then, multiple studies have been conducted to gauge the effectiveness of LF-rTMS on the motor symptoms of PD [286,287,288]. Málly et al. [286] found that only 1 Hz rTMS, not 5 Hz, significantly improved motor scores in PD patients, with the UPDRS scores in those aged ≤65 dropping from 30.3 to 17.8 after one month, and remaining improved at six months (*p* < 0.001). In patients aged > 65, 1 Hz also led to motor and temporary executive function improvements, whereas 5 Hz showed no benefit. Zhuang et al. [288] found that following ten sessions of 1 Hz rTMS, patients showed a significant reduction in motor symptoms, with UPDRS-III scores decreasing by −5.58 ± 3.37 compared to −0.36 ± 1.34 in the sham group (*p* < 0.001), with the benefits lasting up to 3 months. Non-motor symptoms also improved, with the Non-Motor Symptoms Questionnaire (NMSQ) scores being reduced by −1.68 ± 2.11 versus −0.36 ± 1.39 in the sham group (*p* < 0.001); though the effect faded by 3 months, it remained better than sham. Depression, measured by the Hamilton Rating Scale for Depression (HRSD), improved with a maximum score reduction of 4.47 from 13.26, and this effect lasted for at least 3 months. Montreal cognitive assessment (MoCA) scores showed consistent cognitive improvement at all time points, with benefits maintained for up to 6 months post treatment. Lastly, Shukla et al. [287] conducted a meta-analysis that compared LF-rTMS to HF-rTMS, and found slightly better results with the former. Specifically, it revealed that rTMS shows mild but clinically meaningful benefits for motor symptoms in PD, with a pooled UPDRS improvement (*p* < 0.05). LF-rTMS was more consistently effective, especially with higher stimulation doses, while HF-rTMS results were mixed, likely due to variations in protocols. The benefits typically lasted about 6 weeks, and treatment was well tolerated.

### 5.2. Dystonia

Currently, the typical treatment for dystonia includes a combination of botulinum toxin injections, medications, and, in some cases, surgery. However, it is not uncommon for patients to discontinue the use of medications or botox due to ineffectiveness or side-effects [289]. Thus, there exists a need for alternative non-invasive treatment options. Although rTMS has shown therapeutic promise in dystonia, the mechanisms underlying its effects are not yet fully understood [290,291]. Traditionally, motor cortex hyperexcitability has been implicated in the abnormal muscle co-contraction and overflow to adjacent muscles characteristic of dystonia. However, growing evidence points to a broader pathophysiological landscape that includes aberrant plasticity processes and impaired sensorimotor integration [290,292]. Specifically, cortical hyperexcitability in dystonia appears to result from two key abnormalities within the sensorimotor system: a deficit in inhibitory circuit activation and enhanced neural plasticity [293,294]. rTMS, particularly inhibitory protocols, may help rebalance these disturbances by enhancing intracortical inhibition and dampening excessive plasticity. As such, LF-rTMS and continuous theta burst stimulation (cTBS), a patterned protocol that exerts comparable inhibitory effects, have been tested across various cortical targets to reduce dystonic symptoms, with the choice of protocol and target often depending on dystonia subtype. However, according to a literature review by Erro et al. [295], clinical outcomes have been inconsistent.

Earlier investigations provided limited support for rTMS as a reliable intervention in dystonia [23,295]. Many studies focused on the dorsolateral premotor cortex (dPMC) contralateral to the more affected hemisphere. In patients with focal hand dystonia, several sham-controlled trials demonstrated modest symptom relief, particularly improvements in writing and motor control, following LF-rTMS to the dPMC [296,297]. However, the robustness of these findings is limited by methodological constraints, such as single-session protocols or small sample sizes. In a study by Kimberley et al. [298], a five-day stimulation regimen was administered to 17 patients (12 receiving real stimulation and 5 sham). Following the intervention, improvements were observed in both sensory discrimination and functional disability. The Byl–Cheney–Boczai sensory discriminator test showed a significant enhancement in right ring discrimination (*p* = 0.017), lasting up to one week after the second post-test. Additionally, the arm dystonia disability scale (ADDS) revealed a significant reduction in functional impairment (*p* = 0.048). In a sham-controlled study involving 18 patients with focal hand dystonia, Huang et al. [299] delivered daily cTBS sessions to the dPMC over five consecutive days. Real stimulation successfully restored abnormal dPMC–M1 connectivity (*p* < 0.001) as measured by MEPs; however, this effect was only observed after consecutive sessions, with no significant changes detected following the first day of stimulation (*p* < 0.784). More recent work has continued to evaluate rTMS’s efficacy across different forms of dystonia. Hao et al. [300] employed a seven-day rTMS protocol targeting M1 contralateral to the affected upper limb in patients with limb dystonia. The real stimulation group showed significant improvements in muscle tone and stiffness after 1 week (*p* < 0.01), 3 weeks (*p* < 0.01), and 5 weeks (*p* < 0.01), suggesting meaningful motor benefits. Similarly, Poydasheva et al. [301] applied ten sessions of LF-rTMS to the premotor cortex in patients with writer’s cramp, spaced over five days with a two-day break. Encouragingly, clinical improvements persisted for at least one month post treatment (*p* < 0.05), reinforcing rTMS’s potential in focal dystonias.

While these findings collectively highlight the therapeutic value of rTMS, particularly for focal dystonias, the field still lacks robust, long-term studies, especially those targeting cerebellar regions to establish consistent and reproducible efficacy across dystonia subtypes.

### 5.3. Essential Tremor

First-line pharmacologic treatments include propranolol and primidone, with the former being the only FDA-approved treatment for ET [302]. However, 30–50% of patients do not respond adequately to these medications and are instead prescribed alternatives such as anticonvulsants, benzodiazepines, or botulinum toxin [303]. Notably, these treatments are associated with substantial side effects and clinical considerations, including paresthesia, disordered eating, fatigue, cognitive impairment, disorientation, seizures, and muscle weakness, among others [304]. In severe cases of ET, invasive interventions such as DBS may be considered, though these procedures carry their own risks and clinical considerations [304]. Consequently, with approximately half of ET patients not responding adequately to current non-invasive therapies, there is a clear and pressing need to explore alternative non-invasive treatment options. Recently, there has been a growing interest in the potential of rTMS as a therapeutic intervention for ET, particularly through its ability to modulate neural activity within key nodes of the tremor network, including the cerebellum and motor cortex [185,305,306,307,308,309]. The cerebellum, which plays a central role in movement coordination and feedback regulation, is a major component of the CTC pathway—a circuit often implicated in ET pathophysiology and potentially responsive to rTMS modulation [185].

One commonly explored rTMS protocol involves LF-rTMS of the cerebellum. Popa et al. [308] applied bilateral 1 Hz rTMS over the cerebellar cortex for five consecutive days and observed significant reductions in tremor severity, drawing impairment, and functional disability (*p* < 0.006). Moreover, it also re-established the defective information processing in the CTC network (P(Δ|y) > 0.909). Notably, these improvements lasted up to three weeks post-treatment, suggesting a lasting neuromodulatory effect. In contrast, Gironell et al. [309] conducted a single-session cerebellar rTMS intervention and found significant tremor reduction immediately post-stimulation, though the effects returned to baseline within 60 min. This discrepancy underscores the potential importance of repeated rTMS sessions in achieving sustained therapeutic benefits. However, the efficacy of LF-rTMS on tremor suppression remains contentious. A more recent trial by Olfati et al. [185] using an identical 5-day bilateral cerebellar rTMS protocol failed to replicate the previous findings of Popa et al. [308], reporting no significant changes in tremor severity compared to the sham group. These inconsistencies may reflect inter- and intra-individual variability in response to rTMS, or methodological nuances such as coil placement, individual brain anatomy, or differences in baseline tremor characteristics. Regardless, the collective findings emphasize the need for larger, randomized controlled studies to clarify the therapeutic value of cerebellar rTMS in ET. Beyond the cerebellum, cortical targets have also been investigated. Some researchers have attempted stimulation of the left M1 or premotor areas, yet these studies failed to show meaningful improvements in tremor variables in ET, and if they did effect change, it lasted for a very short period or was subclinical in nature [189,196,310]. One study demonstrated that although both the sham and treatment groups showed tremor improvement immediately after 15 daily sessions of pre-SMA LF-rTMS, only the treatment group maintained effects at the 4- and 8-week follow-ups [306]. These findings suggest that the pre-SMA may be a promising and underexplored target for rTMS in ET therapy.

Together, these studies illustrate that the technique is safe and feasible; however, its efficacy for ET remains to be established in large randomized controlled trials. Consequently, future research must refine stimulation parameters, optimize target selection, and account for patient-specific factors through rigorous, large-scale clinical trials.

### 5.4. Alzheimer’s Disease + Mild Cognitive Impairment

There is currently a wide range of medications available to help manage the symptoms and enhance the quality of life for patients with AD. However, these treatments are primarily effective in slowing the progression of cognitive decline, but do not prevent or reverse it [311]. Additionally, up to one-third of patients do not respond to these treatments; hence, there is an urgent need for alternative non-invasive approaches [312]. As AD is characterized by brain atrophy and reduced neuroplasticity, the therapeutic use of rTMS aims to mitigate and, ideally, reverse these effects [313]. Consequently, recent attention has focused on using rTMS in the treatment of AD to promote neuroplasticity and target large-scale circuits that are often impaired in AD, such as the central–executive and hippocampal–parietal networks [314]. Accordingly, the goal of rTMS is to activate long-term potentiation to increase synaptic strength and function [314]. This process can also cause an increase in positive cognitive markers like brain-derived neurotrophic factor (BDNF), which are linked to improved memory and cognition [315]. The beneficial effects of rTMS also extend to MCI specifically in the domain of cognition [300].

Four recent systematic reviews have been conducted on the use of rTMS in AD [316,317,318,319]. The review by Chou et al. [318] included 13 studies with 293 participants, utilizing a meta-analysis approach to evaluate the effects of rTMS on cognitive functions in patients with MCI and AD. The overall pooled effect size of rTMS on cognitive performance was 0.77 (95% CI [0.57, 0.97]), reflecting a medium-to-large improvement in favor of active rTMS compared to the sham (z = 7.69, *p* < 0.0001). Accordingly, significant improvements were seen in memory functions from HF-rTMS over the left DLPFC and LF-rTMS at the right DLPFC and executive performance from HF-rTMS targeting the right inferior frontal gyrus. The effects of treatment were found to last for 4 to 12 weeks post intervention, indicating a potential for sustained cognitive enhancement. The review by Lin et al. [316] evaluated the effects of rTMS on cognitive function in AD patients. A total of 12 studies, comprising 231 patients, were included, focusing on randomized controlled trials. The results indicated a significant improvement in cognition with rTMS compared to the sham rTMS (*p* < 0.0001). Subgroup analyses indicated that stimulating multiple sites, increasing the number of sessions, and extending treatment durations resulted in more significant cognitive improvements. The review by Zhang et al. [317] evaluated the effects of rTMS on global cognitive function in patients with AD. A total of nine studies with 361 patients were included in the meta-analysis, which assessed cognitive changes using the Mini-Mental State Examination (MMSE) and the Alzheimer’s Disease Assessment Scale–Cognitive Subscale (ADAS-Cog). The results showed that rTMS significantly improved cognitive function, with mean differences of 1.82 (*p* < 0.00001) for MMSE and 2.72 (*p* < 0.00001) for ADAS-Cog immediately after treatment, with effects lasting more than 3 months (*p* = 0.0007 for MMSE; *p* = 0.0001 for ADAS-Cog). Subgroup analyses highlighted that HF-rTMS targeting the left DLPFC for over 20 sessions led to the most significant cognitive improvements. Lastly, the review by Wang et al. [319] analyzed the efficacy of rTMS in improving cognitive impairment in patients with AD through a meta-analysis of RCTs. Including 15 trials that involved 240 patients, the results indicated a significant cognitive improvement (standardized mean difference (SMD) of 0.42; *p* = 0.0006) with rTMS compared to sham stimulation. Subgroup analyses showed that rTMS on multiple sites resulted in a greater effect (SMD of 0.47) and that patients receiving more than ten sessions experienced better outcomes. Additionally, they found 20 Hz was more effective than 10 or 1 Hz, and those with higher education levels (≥9 years) showed more significant improvement (SMD of 0.64) compared to those with lower education (<9 years). In summary, rTMS has shown promising potential in terms of improving cognitive function in AD and MCI, particularly with longer treatment durations, multi-site stimulation, and high-frequency protocols. Future research should aim to further refine treatment protocols and explore the long-term benefits of rTMS, as well as identify key patient characteristics that can help predict the most effective outcomes.

**Table 2:** Comparative overview of rTMS parameters in neurological disorders.

Condition	Parameter	Effect + Efficacy
Parkinson’s Disease (PD)	High frequency over M1 (5 Hz).	Short-term improvements in motor function in upper-limb contralateral to stimulation stie (↓ UPDRS-III scores, (*p* = 0.0005) [262].
		Long-term (≤1 month) improvements in motor function (↓ UPDRS-III scores (*p* = 0.0001), ↑ walking speed (*p* = 0.001), and self-assessment scales (*p* = 0.002)) [263].
		Improvements in motor and mood symptoms and LID [23,265,266,267,268,278].
		Resorted excitatory circuits reduced bradykinesia, improved rigidity (↑ ICF and prolonged CSP) [127].
		Improvement in FOG symptoms (improved Standing Start 180° Turn Test and FOG-Q scores, ↓ UPDRS-III scores, and ↓ TUG score (*p* < 0.05)) [277,278].
		Improvement in gait speed, stride length, and variability of gait speed (*p* < 0.05) [280].
		Restored (↑) SICI (*p* = 0.001) [280].
	High frequency over broader motor cortex regions (limb-specific) (>5 Hz).	Improvements in motor symptoms and LID (*p* < 0.001) [267].
	High frequency (>5 Hz) over left-DLPFC.	Improvements in motor function, mood symptoms, and LID [265].
		Improvement in gait and upper limb bradykinesia (*p* < 0.001) [281].
	High frequency over SMA (>5 Hz).	Improvements in LID [265,268].
		Restoration of the basal ganglia–thalamocortical circuitry and motor function (↓ UPDRS-III scores (*p* < 0.0001) [271].
		Improvements in general motor symptoms (↓ UPDRS-III scores (*p* = 0.0001) [272].
		Restored intracortical inhibition (↑SICI, ↑ LICI, and ↑ CSP) [127]
		Improvements in motor symptoms.
	Low frequency over M1, right- and left-DLPFC, and SMA (<1 Hz).	Improvements in motor symptoms (↓ UPDRS-III scores (*p* < 0.05, *p* < 0.0001, *p* < 0.05, *p* < 0.001)) [272,285,286,287,288].
		Improvements in non-motor symptoms (↑ executive function (*p* < 0.0001) ↓ NMSQ score (*p* < 0.0001) ↓ HRSD (*p* < 0.0001)) [286,288].
Dystonia	Low frequency over contralateral dPMC: (≤1 Hz)	Improvements in writing and motor control (focal hand dystonia) (*p* = 0.004) [296,297].
		Improvements in sensory discrimination (improved Byl–Cheney–Boczai sensory discriminator (*p* = 0.017) [298].
		Improvements in functional disability (improved ADDS (*p* = 0.048) [298].
	High frequency (50 Hz) cTBS over contralateral dPMC: (50 Hz)	Restoration of abnormal dPMC-M1 connectivity (*p* < 0.001) [299].
		↑ SICI (*p* < 0.001) [299].
		Clinical improvements in patients with writer’s cramp (*p* < 0.05) [301].
	High frequency over contralateral M1: (10 Hz)	Improvements in muscle tone and stiffness up to 5 weeks (improved MyotonPRO scores (*p* < 0.01) [300].
Essential Tremor (ET)	Bilateral 1 Hz over posterior cerebellar cortex	Improvements in tremor severity, drawing impairment, and functional disability (measured by Fahn–Tolosa–Marin scale)(*p* = 0.006) [308].
		Improvements in information processing in the CTC network (*p*(Δ|y) > 0.909). [308].
	Bilateral 1 Hz 2 cm below inion	Immediate and temporary (60 min) improvements in tremors (decreased Tremor Clinical Rating Scale score (*p* < 0.001)) [309].
	1 Hz over contralateral pre-SMA	Improvements in tremors in treatment groups (dCohen magnitude of 0.49 (moderate effect)) [306].
Alzheimer’s Disease (AD) and Mild Cognitive Impairment (MCI)	Meta-analysis of 13 studies with 293 participants.	Medium-to-large improvements in cognitive performance (effect size 0.77, *p* < 0.0001) [318].
	- High frequency over left-DLPFC and low-frequency over right-DLPFC.	Improvements in memory function (*p* < 0.001) [318].
	- High frequency over the right inferior frontal gyrus.	Improvements in executive performance (*p* < 0.001) [318].
	Analysis of 12 studies with 231 patients (focus on RCTs).	Improvements in cognition with subgroup analysis, showing that increasing stimulation sites, number of sessions, and treatment duration resulted in more significant improvements (*p* < 0.0001).
	Meta-analysis of nine studies with 361 patients.	Immediate improvements in cognition lasting more than three months (improved MMSE (*p* < 0.00001), ADAS-Cog (*p* < 0.0001).
	- High frequency over left-DLPFC for 20 sessions.	Greatest improvements in cognition.
	Meta-analysis of RCTs.	Improvements in cognitive impairment (*p* < 0.0006), which further improves with increasing stimulation sites (SMD 0.47, *p* < 0.0001), number of sessions (>10) (*p* < 0.003), and higher education levels (≥9 years) (SMD 0.64, *p* < 0.001).
		- 20 Hz more effective than 1 Hz and 10 Hz.

ADAS-cog: Alzheimer’s disease assessment scale-cognitive subscale, ADDS: arm dystonia disability scale, CSP: cortical silent period, DLPFC: dorsolateral prefrontal cortex, dPMC: dorsal pre-motor cortex, FOG: freezing of gait, HRSD: Hamilton Rating Scale for depression, ICF: intracortical facilitation, LID: levodopa-induced dyskinesia, M1: primary motor cortex, MMSE: Mini-Mental State Examination, NMSQ: Non-Motor Symptoms Questionnaire, RCTs: randomized control trials, SICI: short-interval intracortical inhibition, SMA: supplementary motor area, UPDRS: Unified Parkinson’s Disease Rating Scale, ↓: reduced, and ↑: increased.

## 6. Conclusions and Future Directions

Transcranial magnetic stimulation has demonstrated considerable utility as both a diagnostic and therapeutic modality in neurological disorders. By evaluating a range of outcome measures, including motor threshold (AMT and RMT), and paired-pulse paradigms such as SICI, LICI, and ICF, TMS enables the in vivo probing of cortical excitability and neurotransmitter-specific circuitry. This neurophysiological profiling has proven effective in distinguishing Alzheimer’s disease and mild cognitive impairment from healthy controls and other neurodegenerative conditions, with multiple studies reporting reproducible abnormalities across these measures in AD and MCI populations. Collectively, these findings suggest that TMS-based biomarkers may offer meaningful sensitivity and specificity for early or differential diagnosis. If similarly rigorous, large-scale investigations were extended to other neurological disorders, including Parkinson’s disease, dystonia, and essential tremor, TMS could be further developed as a complementary diagnostic tool in settings where clinical assessment and conventional imaging are often inconclusive, particularly in early disease stages. Importantly, however, progress in this area is currently limited by substantial methodological heterogeneity across studies, including variability in the stimulation parameters, cortical targets, coil types, outcome measures, and interstimulus intervals used in paired-pulse protocols. This methodological heterogeneity contributes to variability and limits interpretability across studies, leading to inconsistent findings even for well-characterized outcome measures such as SICI in Parkinson’s disease. Greater standardization of these methodologies is essential to improve reproducibility and cross-study comparability. In parallel, rTMS has demonstrated therapeutic potential across a spectrum of neurological disorders, including Parkinson’s disease, dystonia, essential tremor, Alzheimer’s disease, and mild cognitive impairment. Both high- and low-frequency stimulation protocols, when applied to disease-relevant cortical targets, have shown varying degrees of success in modulating pathological neural circuits and improving motor and non-motor symptoms through mechanisms linked to cortical excitability, synaptic plasticity, and neurotransmitter modulation. Despite these encouraging results, consensus on optimal treatment parameters remains lacking, in part due to substantial heterogeneity across rTMS protocols, as well as more common methodological limitations such as small sample sizes, insufficient blinding/control, and short follow-up durations. Future progress will require harmonized, multi-center rTMS trials, the integration of multimodal approaches such as TMS–EEG to capture network-level effects, and longitudinal designs to assess the durability and disease-stage specificity of responses. For example, standardizing the interstimulus intervals (ISIs) used in paired-pulse paradigms according to current consensus is essential to improve cross-study comparability and ensure that findings are interpretable and generalizable. While TMS is not yet a first-line therapy, it holds considerable promise as an adjunct for patients who are unresponsive to pharmacological interventions, with the potential to delay or reduce reliance on invasive procedures. This review seeks to underscore the substantial potential of TMS alongside the limitations imposed by methodological heterogeneity, and to support its progression toward standardized, mechanistically grounded, and clinically integrated use.

## Data Availability

No new data were created or analyzed in this study. Data sharing is not applicable to this article.
